# Factors Influencing the Interactions in Gelatin/Hydroxyapatite Hybrid Materials

**DOI:** 10.3389/fchem.2020.00489

**Published:** 2020-06-11

**Authors:** Zixin Zhang, Kexin Li, Weixian Zhou, Jin'ge Gu, Ying Liu, Charles C. Han, Shanshan Xu

**Affiliations:** ^1^Institute for Advanced Study, Shenzhen University, Shenzhen, China; ^2^State Key Laboratory of Polymer Physics and Chemistry, Joint Laboratory of Polymer Science and Materials, Beijing National Laboratory for Molecular Sciences, Institute of Chemistry, Chinese Academy of Sciences, Beijing, China; ^3^Key Laboratory of Medicinal Chemistry and Molecular Diagnosis of Ministry of Education, College of Chemistry and Environmental Science, Hebei University, Baoding, China; ^4^CAS Key Laboratory for Biomedical Effects of Nanomaterials and Nanosafety & CAS Center for Excellence in Nanoscience, National Center for Nanoscience and Technology of China, Beijing, China

**Keywords:** hydroxyapatite, gelatin, penetrating growth, interface force, nucleation mechanism

## Abstract

The most severe problem in bone regeneration is the defect in the interface. We prepared four types of implantation scaffolds of crosslinked gelatin (GE)/hydroxyapatite (HAp) to study the factors influencing interface interactions, they are film-crosslinked GE scaffold, gel-crosslinked GE scaffold, solid-crosslinked GE/HAp scaffold and gel-crosslinked GE/HAp scaffold. HAp could penetrate the entire GE matrix completely in four successive steps: physical preparation of a gel; chemical crosslinking; incubation in modified simulated body fluid (m-SBF) and freeze-drying. The penetrative nucleation and growth of HAp and the influencing factors in the GE matrix were investigated to ameliorate the interface interactions between organic and inorganic layers. During development of penetrative nucleation and growth, a tight connection was built between organic and inorganic layers, B-type carbonated HAp was formed after incubation with m-BSF, and the apatite content could be controlled. In summary, enhanced interface relies on not only the pre-seeded hydroxyapatite (HAp) as crystal nuclei but also the sufficient space for ions with high concentration to diffuse in.

## Introduction

Functional substitutes for the repair and regeneration of bone have garnered increasing attention (Pina et al., 2015; Habraken et al., 2016) because of undesirably high failure rates and implant loss. The interface interaction (Bauer et al., 2013) is very important to solve the main complications, aseptic loosening (Laquerriere et al., 2003; Buchanan and Goodfellow, 2007), and infection in surrounding joints, which result from the particles produced from prosthetic interfaces (Bader et al., 2004; Grandjean-Laquerriere et al., 2004; Liu-Snyder and Webster, 2008; Raphel et al., 2016). One can analogize bone structure to that of “steel” and “cement.” That is, the nucleation mechanism of the cement and the factors inducing the cement to penetrate steel are important to ensure tight combination of steel with cement, and reduction of the interface force. The research mainly focuses on the interface interaction and crystal growth mechanism of organic and inorganic hybrid materials.

Hydroxyapatite (HAp) is the best choice as “cement” because the human bone contains ~70% HAp (Habraken et al., 2016; Farokhi et al., 2018). HAp has good biocompatibility and osteo-conductivity and can bind tightly to bone tissue (Jee et al., 2010; Roohani-Esfahani et al., 2010; Fu et al., 2012; Liu et al., 2019). Gelatin (GE) is chosen as “steel” because it is the hydrophilic hydrolysate of bone collagen, and can be converted readily to a hydrogel state if cooled to a critical gel transition temperature. Water and ions can diffuse freely in the gaps of the hydrogel network, which provide the essential environment for internal bonding.

Finding a way to increase the interaction between GE and HAp is controversial (Kim et al., 2008; Liu et al., 2009; Kolambkar et al., 2011; Amosi et al., 2012; Frohbergh et al., 2012; Li et al., 2012). Existing research interests can be generally divided into three categories: pure physical bonding, adding chemicals and mechanism related. The coating method could cause the formation of unstable layers (Tapsir and Saidin, 2016). Lamination technology has been used to glue porous layers (Azami et al., 2010). Although some of these physical bonding methods can reach the mechanical strength of spongy cancellous bone, subsequent aging, and long-term implantation experiments have not been performed, so the clinical effect is uncertain.

Some researchers have added chemicals to improve interface bonding and mechanical strength (Ryu et al., 2010; Tapsir and Saidin, 2016). Surface modification has been used to improve interaction at the inorganic–organic interface (Li et al., 2007; Rao et al., 2014). The importance of the nucleation and growth of HAp on the matrix has also been considered (Cui et al., 2010; Zahn, 2017) as well as the factors influencing nucleation, for example, the relationship between temperature or gelatin concentration and crystal size (Chang et al., 2003, 2006), the functional groups on the substrate (Cui et al., 2010), and presence of a matrix (Katti et al., 2006; Bhowmik et al., 2007). Studies have pointed out that ions are combined with organic matrices as “seeds” to lead to a stable crystal orientation (Sato, 2007). The early and uniform apatite formed on the cement surface showed a stable interface and high bioactivity (Masahiko Kobayashi et al., 1997).

We assumed that the penetration of crystals into the matrix could weaken the interfacial force. Four implantation scaffolds of GE/HAp were built to study the factors influencing the nucleation and growth of HAp in GE scaffolds. These factors were imitation of *in situ* nucleation, pre-planting of crystal seeds, and different substrate macromolecular chain interspaces. A potential strategy, promoting *in situ* heterogeneous nucleation by adding HAp seeds in advance, could promote crystallization inside and increase the interactions between the HAp and GE matrix. This research can give a guidance for organic-inorganic hybrid materials field and provide a reference when others want to investigate deeply. We also show the possibility of solving interface force problem and improving existing products in hybrid materials application.

## Materials and Methods

### Materials

GE (type B, from cow hides, 110–130 Blooms) was purchased from Thermo Scientific (Waltham, MA, USA). HAp was obtained from J&K Scientific (Beijing, China). Glutaraldehyde (25% water solution) was purchased from Shantou Xilong Chemicals (Shantou, China). Sodium carbonate and 1,4-dioxane were obtained from Beijing Chemical Works (Beijing, China). Sodium chloride, potassium phosphate dibasic trihydrate, 4-(2-hydroxyethyl) piperazine-1-ethanesulfonic acid and calcium chloride (anhydrous) were purchased from Sigma–Aldrich (Saint Louis, MO, USA). Sodium bicarbonate, potassium chloride, magnesium chloride hexahydrate, sodium hydroxide, and sodium sulfate were obtained from Sinopharm Chemical Reagents (Shanghai, China).

### Preparation of Scaffolds

**Figure 1:**
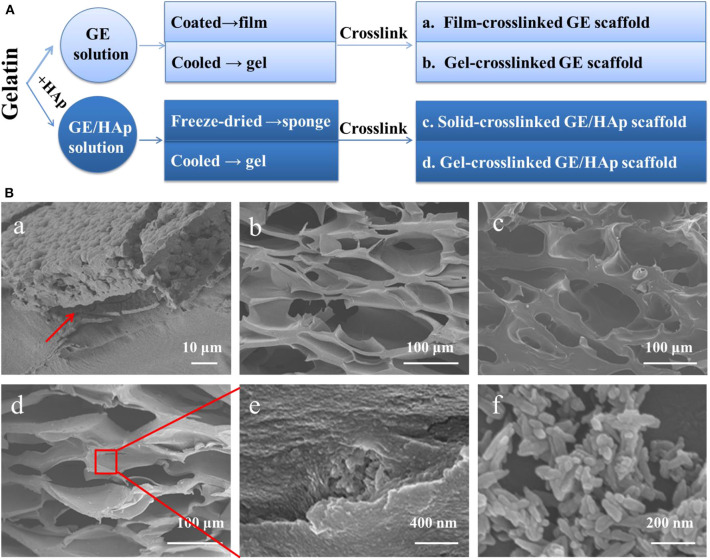
Preparation of GE/HAp scaffolds. Coating film cause a separation interface; all porous scaffolds have suitable pore sizes to conduct bone regeneration. **(A)** Preparation method of different scaffolds. **(B)** SEM micrographs of different scaffolds. **(B**a**)** Cross-section of HAp grown on GE film after 14 days. Red arrow indicates the separation interface. **(B**b**)** Cross-section of a porous gel-crosslinked GE scaffold before incubation. **(B**c**)** Cross-section of a porous solid-crosslinked GE/HAp scaffold before incubation. **(B**d**)** Cross-section of a porous gel-crosslinked GE/HAp scaffold before incubation. **(B**e**)** Aggregates of HAp in a GE matrix. **(B**f**)** Commercial nano HAp.

Four types of scaffolds were fabricated using four procedures (Figure 1A) as follows.

Film-crosslinked GE scaffolds were prepared. Briefly, GE (1 g) was added to 10 mL of ultrapure water with vigorous magnetic stirring for 2 h at 50°C. The solution was coated in a 6-mm petri dish to form a cast film which was chemically crosslinked in a 1% glutaraldehyde solution (90/10, (*v/v*), 1,4-dioxane/water) for 6 h at 4°C. To maintain the microstructure and prevent the dissolution and swelling of the GE matrix, 1,4-dioxane/water (90/10) solvent was selected (Liu et al., 2009).

Gel-crosslinked GE scaffolds and gel-crosslinked GE/HAp scaffolds were prepared. Briefly, for gel-crosslinked GE/HAp scaffolds, HAp (0.05 g) was immersed in 10 mL of ultrapure water with ultrasonic agitation for 30 min first. Then, GE (1 g) was added to this solution with vigorous magnetic stirring for 2 h at 50°C. Then, the mixture was poured into a 6-mm petri dish and cooled rapidly to 4°C to induce gelation. Afterwards, the scaffolds were chemically crosslinked in 1% glutaraldehyde solution for 6 h at 4°C. Gel-crosslinked GE scaffolds without seed crystal HAp was prepared by the same procedure.

Solid-crosslinked GE/HAp scaffolds were prepared. Briefly, HAp (0.05 g) was immersed in 10 mL of ultrapure water with ultrasonic agitation for 30 min. Then, GE (1 g) was added to this solution with vigorous magnetic stirring for 2 h at 50°C. Then, the mixture was poured into a 6-mm petri dish, frozen at −20°C for 24 h, transferred to a lyophilizer, and freeze-dried for 3 days. Afterwards, the scaffolds were chemically crosslinked in 1% glutaraldehyde solution (90/10 (*v/*v), 1,4-dioxane/water) for 6 h at 4°C.

All scaffolds were washed thrice with ultrapure water. This was followed by incubation in pure water (which was changed every 6 h) for 24 h to remove the remaining glutaraldehyde and 1,4-dioxane.

### Growth of HAp Crystals in Different Scaffolds

Scaffolds were cut into pieces (10 × 10 mm^2^) and incubated in modified simulated body fluid (m-SBF) at 37°C, which was prepared as reported previously (Oyane et al., 2003). In the first 3 days, 1× m-SBF was used to incubate the scaffolds. Then, the solution was replaced by 1.5× m-SBF and changed every 2 days. After incubation in m-SBF for 7, 14, or 21 days, parts of the scaffolds were removed from m-SBF and rinsed several times with ultrapure water. Afterwards, the scaffolds were frozen at −20°C for 24 h, freeze-dried for 3 days, and preserved in a vacuum desiccator. The scaffolds were named “film-crosslinked GE/apatite,” “gel-crosslinked GE/HAp/apatite,” “gel-crosslinked GE/apatite,” and “solid-crosslinked GE/HAp/apatite.”

### Characterization of Different Scaffolds

The morphology of the surfaces, fractured surfaces, and pore structures of scaffolds were observed using scanning electron microscopy (SEM) employing a JSM-6700F (JEOL, Tokyo, Japan) system.

X-ray diffraction (XRD) analyses were undertaken using an X-ray diffractometer (Empyrean; PANalytical, Almelo, the Netherlands). Scaffold surfaces were scanned over a 2θ range from 5° to 60° at 2°/min.

Fourier-transform infrared (FTIR) spectroscopy using a Tensor 27 (Bruker, Billerica, MA, USA) system was used to characterize the structure of the formed apatite in scaffolds. For attenuated total reflection (ATR) analyses, spectra were recorded at 4,000–650 cm^−1^ at a resolution of 4 cm^−1^ and 32 scans. IR spectroscopy was done at 4,000–400 cm^−1^ at a resolution of 4 cm^−1^ and 32 scans.

To determine the degree of mineralization in scaffolds, thermogravimetric analysis (TGA) was done with a thermogravimetric analyzer (Pyris 1 TGA; PerkinElmer, Waltham, MA, USA). Experiments were undertaken at a heating rate of 5°C/min under air, and the temperature range was 50–700°C.

Energy dispersive X-ray spectroscopy (EDS) was carried out on an EDAX (Mahwah, NJ, USA) system to evaluate apatite distribution in gel-crosslinked GE/HAp/apatite scaffolds. Ca^2+^ distribution can represent the apatite distribution. Hence, Ca^2+^ distribution was analyzed by EDS during SEM with an acceleration voltage of 15 kV.

The variation in Ca^2+^ concentration of m-SBF on the first day of incubation (which denotes the mechanism of HAp nucleation) was measured using inductively coupled plasma-atomic emission spectroscopy (ICP-AES) employing an ICPE-9000 (Shimadzu, Tokyo, Japan) system.

## Results and Discussion

Since the implantation of artificial bone or bone cement is porous structure, gel GE scaffolds with or without HAp were crosslinked to simulate the natural bone structure and to find out the influence of HAp. To directly investigate the interaction of GE and HAp on the interface, a film of GE was crosslinked as comparison. Moreover, the mineralization of scaffold can be affected not only by the interface interaction of hybrid material and the crystal nuclei but also by the supplementary of crystal ions. The solid scaffold was crosslinked after freeze-drying to modify the ion diffusion channel.

### Mineralization of Scaffolds Was Affected by the Preparation Steps

Different preparation designs lead to different mineralization process. For gel-crosslinked scaffolds, chemical crosslinking took place immediately after the physical gels had been formed. The large interspaces between networks of gels were retained, which provided many channels for the diffusion of water and ions. However, a freeze-drying procedure took place, and a porous structure was formed first for the solid-crosslinked GE/HAp scaffold. Simultaneously, the interspaces between GE molecular chains were compressed, and the pore walls became solid instead of loose. These differences in microstructure could result in different appearances of mineralization within scaffolds, which is essential for bone regeneration.

### Porous Scaffolds Before Incubation had Suitable Apertures for Bone Regeneration

For bone functional substitutes, structures that facilitate bone regeneration are necessary. A thin GE film led to a separation interface (Figure 1Ba). Direct coating produced marked interfacial delamination and weak internal forces. The pore sizes of the other three porous scaffolds (Figure 1Bb–e) ranged from 100 to 300 μm, which were all suitable to conduct bone regeneration. Scaffolds incubated in m-SBF possessed a similar porous structure. Figure 1Bf shows the micromorphology of HAp: it was rod-like, the length ranged from 40 to 200 nm, and the width ranged from 30 to 50 nm. Thanks to vigorous stirring during preparation of the GE/HAp suspension, HAp was dispersed uniformly in the GE matrix before and after gelation, providing indispensable pre-conditions for subsequent nucleation growth.

### Gel-Crosslinked GE/HAp Scaffolds Induced Apatite Nucleation

**Figure 2:**
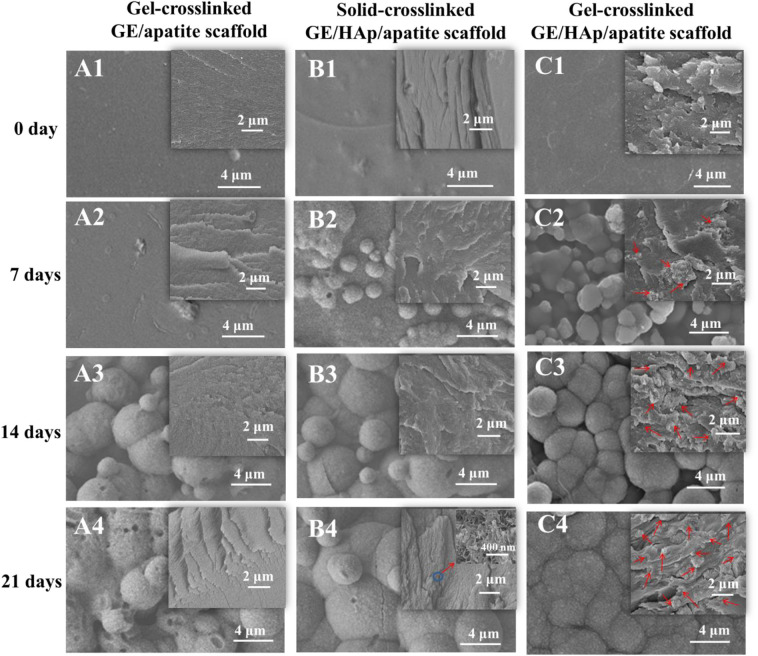
SEM micrographs of different scaffolds with various incubation time. Gel-crosslinked GE/HAp scaffolds induced apatite nucleation. The surface and cross-section morphologies of **(A)** gel-crosslinked GE/apatite scaffolds, **(B)** solid-crosslinked GE/HAp/apatite scaffolds, **(C)** gel-crosslinked GE/HAp/apatite scaffolds incubated in m-SBF for 0 day (A1, B1, C1), 7 days (A2, B2, C2), 14 days (A3, B3, C3), and 21 days (A4, B4, C4) were detected using SEM. The insets are the cross-section morphologies, and red arrows indicate newly grown apatite.

To investigate the apatite distribution and nucleation process in scaffolds, SEM was used to detect surface and cross-section morphologies. Gel-crosslinked GE/apatite, solid-crosslinked GE/HAp/apatite, and gel-crosslinked GE/HAp/apatite scaffolds are shown in Figure 2. Before incubation in m-SBF, the surfaces of the scaffolds were smooth, and only a few HAp aggregates were observed on the surfaces of gel-crosslinked GE/HAp and solid-crosslinked GE/HAp scaffolds. Obvious differences occurred after incubation in m-SBF for 7 days: many apatite semi-spheres or spheres were formed on the surface of gel-crosslinked GE/HAp/apatite scaffolds. Some areas remained uncovered on the surface of solid-crosslinked GE/HAp/apatite scaffolds. Little disk-like apatite was formed on the surface of gel-crosslinked GE/apatite scaffolds. The different surface morphologies of these three scaffolds illustrated that apatite spheres could be induced readily by pre-seeded HAp as the nucleation site rather than the carboxyl group on the GE surface. As incubation time increased, the surface morphologies became similar, and all three scaffold types became covered by apatite spheres.

Cross-section morphologies are shown in the insets of Figure 2. Before incubation in m-SBF, only a few aggregates of rod-like HAp were observed in gel- and solid-crosslinked GE/HAp scaffolds. After incubation for 7 days, a few irregular apatite spheres with sheet-like substructures were found in the GE matrix of gel-crosslinked GE/HAp/apatite scaffolds. However, no apatite was observed in gel-crosslinked GE/apatite scaffolds, whereas some rod-like HAp aggregates were found in solid-crosslinked GE/HAp/apatite scaffolds. With increasing incubation time, increasing numbers of irregular apatite spheres were formed in the GE matrix of gel-crosslinked GE/HAp/apatite scaffolds. The structure of the spheres was similar to the structure of spheres observed on the surface of the scaffold, suggesting that the apatite grew and penetrated the GE matrix. Substantial changes were not observed in the other two scaffold types.

The large interspaces between gel networks in gel-crosslinked scaffolds provided channels for the diffusion of water and ions. Two requirements were needed to grow apatite into the GE matrix: (a) the matrix should be loose with large interspaces between macromolecular chains or networks to ensure free diffusion of water and ions; (b) there should be sites of strong nucleation within the matrix (these would be more competitive than carboxyl groups on the scaffold surface) to induce penetrative growth.

### The Content of Newly Grown Carbonated HAp (c-HAp) Was Controllable

**Figure 3:**
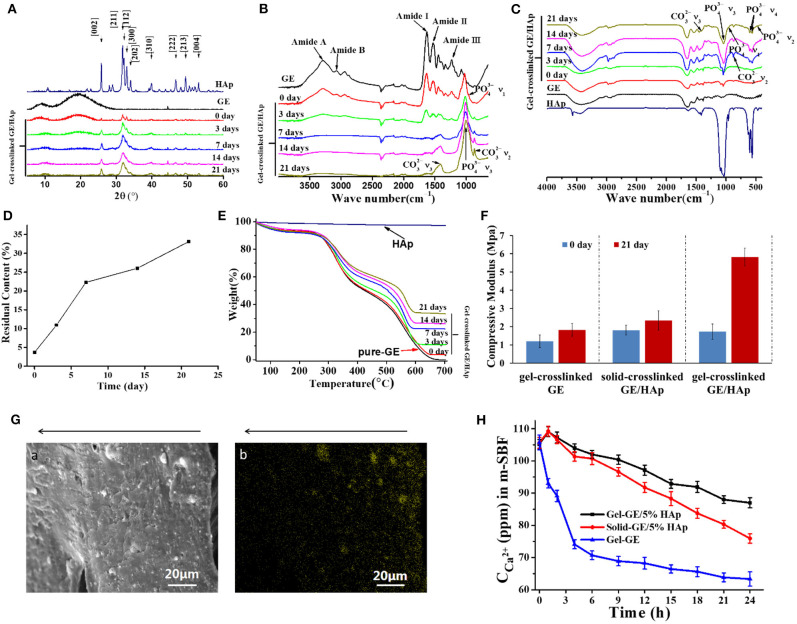
Characterization of GE/HAp scaffolds. Newly grown c-HAp was penetrating into Gel-crosslinked GE/HAp scaffolds, leading to modulus increased, and the content was controllable. **(A)** XRD patterns, **(B)** ATR-FTIR spectroscopy, **(C)** FTIR spectroscopy, and **(D,E)** TGA of gel-crosslinked GE/HAp scaffolds after incubation in m-SBF for various times. **(F)** Compressive modulus of different scaffolds before and after m-SBF incubation. **(G)** (a) SEM micrograph and (b) Ca^2+^ EDS diagram of a cross-section of a gel-crosslinked GE/HAp/apatite scaffold (21 days). The arrow indicates the direction from surface to the inner region. **(H)** ICP diagram of Ca^2+^ concentration in m-SBF after incubation of different scaffolds for various times.

After investigation about morphology and nucleation, crystal properties need to be confirmed. The surfaces of gel-crosslinked GE scaffolds, gel-crosslinked GE/HAp/apatite scaffolds, and rod-like HAp powder were assessed by XRD analyses (Figure 3A). The XRD pattern of HAp was documented in all gel-crosslinked GE/HAp/apatite scaffolds, and was in good agreement with that of HAp powder. The characteristic peaks of other apatite types were not observed, suggesting that the apatite obtained by incubating scaffolds in m-SBF was pure HAp. However, grown HAp showed broad and weak diffraction peaks compared with those of HAp powder, which implied that HAp was poorly crystallized. XRD patterns also showed variation in the HAp layer covering the surfaces of the scaffolds with incubation time. Initially, there were two broad diffraction peaks corresponding to GE, but the diffraction peaks of GE became progressively weaker. This result indicated that the amount and thickness of the HAp layer increased with incubation time, which agreed with the SEM results shown in Figure 2.

The FTIR spectra of gel-crosslinked GE/HAp/apatite scaffolds incubated in m-SBF for various times are shown in Figures 3B,C. According to ATR-FTIR spectroscopy, GE and gel-crosslinked GE scaffolds exhibited major peaks in amide regions. The amide-I band (representing C=O stretching vibration/hydrogen bonding coupled with COO–) was observed at 1,630 cm^−1^. The amide-II band (attributed to the bending vibration of N-H groups and stretching vibration of the C-N group) was observed at 1,530 cm^−1^. The amide-III band (illustrating the in-plane vibration of C-N and N-H groups of bound amide or vibrations of CH_2_ groups of glycine) was noticeable at 1,232 cm^−1^. In addition, the amide-A peak (representing NH– stretching coupled with hydrogen bonding) was observed at 3,291 cm^−1^, and the amide-B peak (corresponding to the asymmetric stretching vibration of =C-H and –${\text{NH}}_{3}^{+}$) was noticeable at 3,072 cm^−1^ (Muyonga et al., 2004; Jridi et al., 2013; Nagarajan et al., 2013; Tongnuanchan et al., 2013). However, for gel-crosslinked GE/HAp/apatite scaffolds incubated in m-SBF for various times, bands corresponding to GE became progressively weaker with increasing incubation time. From 7 days, the bands corresponding to GE disappeared, which implied that the scaffold surfaces were covered completely by HAp.

With increasing incubation time, the peaks indicating c-HAp growth on scaffold surfaces were also observed (Figure 3C). In c-HAp, some ${\text{PO}}_{4}^{3-}$ sites of apatite were substituted by ${\text{CO}}_{3}^{2-}$: this is a similar structure to the HAp found in bone. The peaks at 468, 565 and 601, 962, and 1,048 cm^−1^ corresponded to the ν_2_, ν_4_, ν_1_, and ν_3_ vibrations of ${\text{PO}}_{4}^{3-}$ of HAp, respectively. Simultaneously, the ν_3_ and ν_2_ vibrations of ${\text{CO}}_{3}^{2-}$, at 1,414 and 870 cm–^1^, respectively, were observed. Other vibrations of ${\text{CO}}_{3}^{2-}$ were not observed, suggesting that the c-HAp formed in scaffolds and on scaffold surfaces involved B-type substitution. From 0 day to 21 days, the ratio of intensity $I_{PO_{4}^{3-}\left(v_{3}\right)}$/*I*_*AmideI*_ increased, indicating that the total amount of c-HAp increased with incubation time.

TGA was done to measure the content of c-HAp grown in gel-crosslinked GE/HAp/apatite scaffolds (Figure 3E). HAp is slightly soluble in water, so part of HAp was dissolved in water during scaffold preparation. Thus, the actual content of incorporated HAp was 3.7%. With increasing incubation time, the content increased to 33.1% on day 21 (Figure 3D). Apparently, the content of newly grown carbonated HAp was controllable.

### The Modulus Increased as a Result of the Penetrative Growth of c-HAp

Modulus as an important measure of mechanical properties is of great significance for bone implant substitutes. After incubation in m-SBF for 21 days, the gel-crosslinked GE/HAp/apatite was hard after removal from m-SBF. However, the gel-crosslinked GE/HAp with the commercial HAp we purchased (33%) was soft. This large difference in the modulus between the scaffolds incubated or not incubated in m-SBF (Figure 3F) also reflected better transformation from commercial HAp to the grown c-HAp. Moreover, the scaffolds will be used under wet conditions, so scaffolds prepared by this procedure will be appropriate for bone substitution.

### Newly Grown c-HAp Penetrated Into Scaffolds

A large area of the scaffold was scanned by EDS to evaluate HAp distribution in the gel-crosslinked GE/HAp/apatite scaffold incubated in m-SBF for 21 days. The EDS diagram for Ca^2+^ is shown in Figure 3Ga,b and Figure 3Ga is the SEM micrograph of Figure 3Gb. The bright yellow spots in Figure 3Gb represent the distribution of Ca^2+^ and the distribution of Ca^2+^ represented the distribution of c-HAp. The arrows above the images indicate the inner side of the scaffold. Figure 3G shows the inner region of the scaffold, so c-HAp spheres penetrated the entire scaffold.

We hypothesized that the c-HAp distribution was related to the diffusion of Ca^2+^ and ${\text{HPO}}_{4}^{2-}$. Although ions could diffuse in GE gel, the diffusion rate of ions decreased from the outer surface to inner regions of the scaffolds because GE molecules restricted the diffusion of ions. Therefore, the concentration of Ca^2+^ and ${\text{HPO}}_{4}^{2-}$ decreased from the outer surface to inner parts of the scaffold. The concentration of Ca^2+^ and ${\text{HPO}}_{4}^{2-}$ influenced the nucleation and growth of c-HAp directly and, finally, determined the local content, and distribution of c-HAp in various regions.

In summary, a large diffusion space, nucleation induction in site, and a sufficiently high ion concentration were necessary for HAp-penetrative growth. To determine the mechanisms of nucleation and growth of HAp in gel-crosslinked GE/HAp scaffolds, ICP measurements were undertaken on Ca^2+^ in m-SBF (Figure 3H). Some HAp exposed on the surface was dissolved in m-SBF after 1 h of incubation, so the Ca^2+^ concentration in gel-crosslinked GE/HAp scaffolds and solid-crosslinked GE/HAp scaffolds did not decrease: it increased. Until after 4 h of incubation, the Ca^2+^ concentration was lower than that in m-SBF before incubation of the scaffold. Subsequently, the Ca^2+^ concentration continued to decrease, suggesting that Ca^2+^ was consumed for the nucleation and growth of HAp. However, the Ca^2+^ concentration in m-SBF after incubation of gel-crosslinked GE/HAp was always higher than that of solid-crosslinked GE/HAp scaffolds. These observations could have been because water and ions could diffuse in gel-crosslinked GE/HAp scaffolds because of the large interspaces between networks. Thus, except for HAp dissolved from the surface, HAp in the inner parts of the gel-crosslinked GE/HAp scaffold were also partly dissolved to supply the Ca^2+^ in m-SBF, which led to a higher concentration of Ca^2+^. However, for gel-crosslinked GE, the Ca^2+^ concentration decreased from the beginning to the end because a complementary source of Ca^2+^ was not available.

### Proposed Mechanism of Nucleation

**Figure 4:**
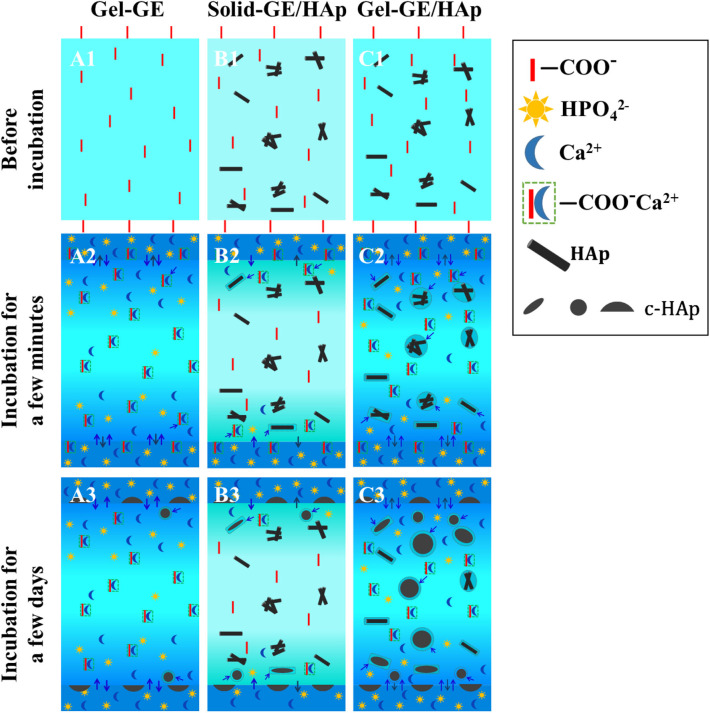
The scheme represents the nucleation-and-growth mechanisms of **(A)** gel-crosslinked GE scaffolds, **(B)** solid-crosslinked GE/HAp scaffolds, **(C)** gel-crosslinked GE/HAp scaffolds. The loose structure matrix with larger interspaces between macromolecular chains, stronger inducive nucleation sites, and a high concentration of ions were play important roles in mechanism conception.

Based on the results obtained above, we put forward the following hypotheses for the crystallization mechanism in gel-crosslinked GE scaffold, solid-crosslinked GE/HAp scaffold and gel-crosslinked GE/HAp scaffold. As shown in Figure 4. Figure 4A represents the gel-crosslinked GE scaffold. When the scaffold was incubated in m-SBF, water and ions diffused in the scaffold. Due to hindrance by networks of molecular chains, fewer ions diffused in the inner regions than those in the outer regions (which is represented by a gradual shading to lighter colors). Then, some Ca^2+^ bound to –COO^−^ to form nucleation sites. Apart from a few HAp semi-spheres and spheres formed on (or very close to) the surfaces, no HAp spheres formed in the inner regions of the scaffolds. Due to hindrance by networks of chains of GE molecules, the concentration of diffused Ca^2+^ and ${\text{PO}}_{4}^{3-}$ was not sufficient to nucleate and grow HAp.

Figure 4B represents the solid-crosslinked GE/HAp scaffold. The pores were formed by freeze-drying before crosslinking, in which the interspaces between chains of GE molecules were forced out. The solution used for crosslinking contained only 10% water, so swelling was prevented in the crosslinking procedure, which led to the pore walls remaining solid. Water and ions could not diffuse in the scaffold, which is described by a much lighter color than Figures 4A,C. Thus, HAp could nucleate and grow only on the surface or the region very close to the surface.

Figure 4C represents the gel-crosslinked GE/HAp scaffold. As mentioned above, some HAp in the inner regions of the scaffold was dissolved in m-SBF, and most of the dissolved Ca^2+^ and ${\text{PO}}_{4}^{3-}$ was distributed in a small region close to surfaces of HAp rods, and formed a high-concentration region. The dissolved Ca^2+^ and ${\text{PO}}_{4}^{3-}$ combined with those diffused from m-SBF to form a supersaturated region for the nucleation and growth of HAp. However, the nucleation sites formed by Ca^2+^ binding to –COO^−^ could be divided to two types: (i) close to the scaffold surfaces and (ii) in the inner regions of the scaffold. For (i), the concentration of Ca^2+^ and ${\text{PO}}_{4}^{3-}$ was supersaturated around the previous nucleation sites because Ca^2+^ and ${\text{PO}}_{4}^{3-}$ could diffuse readily to this region and some Ca^2+^ and ${\text{PO}}_{4}^{3-}$ were from dissolved HAp rods, so HAp could nucleate and grow in this region. For (ii), the concentration of Ca^2+^ and ${\text{PO}}_{4}^{3-}$ was not sufficient to nucleate and grow HAp.

## Conclusions

GE/HAp scaffolds with HAp penetrating into the scaffold were prepared. The mechanisms of the nucleation and growth of HAp in the inner regions of the scaffolds were investigated. The scaffolds were fabricated successively by physical preparation of hydrogels, immediate chemical crosslinking, incubation in m-SBF, and lyophilization. c-HAp (B-type) was incorporated into the scaffolds, and c-HAp was very similar to the inorganic mineral found in bone. Fabrication using this method enabled the compressive modulus to be increased substantially, and the penetrative growth could be attributed to good interactions between HAp and GE macromolecules to solve the problem of interfacial forces. Also, the mechanisms of the nucleation and growth of HAp were clarified. That is, the loose structure matrix with larger interspaces between macromolecular chains, stronger inducive nucleation sites, and a high concentration of ions were needed. However, the unevenness of the distribution remained because the HAp crystal could grow on the surface of the matrix indeed easier than penetrate into the interior of the scaffolds. Therefore, a scaffold with a gradient of pore sizes (larger in the inner side and relatively smaller in the region in contact with the culture medium) or a gradient of seed distribution could alleviate this problem. Furthermore, all the experiments in this study were carried out *in vitro*. The supplementary of sufficient Ca^2+^ ions and the mechanical compliance of the scaffold with natural bone tissues need further investigations.

## Data Availability Statement

The original contributions presented in the study are included in the article/supplementary material, further inquiries can be directed to the corresponding author/s.

## Author Contributions

SX and CH conceived and designed the study. ZZ, KL, and WZ carried out the experiments. ZZ, KL, WZ, and JG analyzed the data. KL and ZZ wrote the manuscript. YL offered a *critique* of the manuscript for important intellectual content. All authors contributed to the final version of the manuscript and approved it for submission.

## Conflict of Interest

The authors declare that the research was conducted in the absence of any commercial or financial relationships that could be construed as a potential conflict of interest.
